# Editorial: New progress in the treatment of bone and soft tissue tumors

**DOI:** 10.3389/fphar.2024.1430844

**Published:** 2024-05-23

**Authors:** Duoyi Zhao, Jiachen Yu, Chengbin Ma, Xiaoyu Xu, Zhiyu Zhang

**Affiliations:** ^1^ Department of Orthopedics, The Fourth Affiliated Hospital of China Medical University, China Medical University, Shenyang, Liaoning, China; ^2^ McKay Orthopedics Research Lab, University of Pennsylvania, Philadelphia, PA, United States

**Keywords:** bone and soft tissue tumors, osteosarcoma, chemotherapy, immunotherapy, nanotechnology engineering, clinical transformation

Bone and soft tissue tumors, constitute a heterogeneous group of neoplasms that frequently affect the young. These tumors are highly aggressive, often metastasizing early in the disease’s progression, posing a significant challenge to orthopedic oncologists. Treatment strategies for these tumors have evolved, with a mainstay approach combining surgery, chemotherapy, and more recently, the introduction of targeted therapies and immunotherapy. Over the past decade, there have been significant advancements in the molecular understanding of these tumors, leading to the development of innovative therapeutic strategies that hold promise for improving patient outcomes and potentially modifying the prognosis of these malignancies.

This study encompassed a total of 10 articles, comprising 4 original research articles, 5 review articles, and 1 case report.

Chen-xi et al. conducted thorough research into the role of long non-coding RNA PRR7-AS1 in the development of bone sarcoma and evaluated its potential as a therapeutic target. The team discovered that PRR7-AS1 interacts with the protein RNF2, thereby inhibiting the translation of the downstream gene MTUS1, which in turn promotes the proliferation and migration of bone sarcoma cells. Furthermore, the researchers found that the knockdown of PRR7-AS1 significantly suppressed the *in vitro* proliferation and the *in vivo* growth and metastasis of bone sarcoma cells. These findings underscore the oncogenic role of PRR7-AS1 in bone sarcoma and suggest its potential as a novel target for diagnosis and treatment.

Miwa et al. reported on a case of pulmonary giant cell tumor of the bone (GCTB) that was inoperable and successfully treated with Denosumab. The case involved a 49-year-old male who had undergone two surgeries for GCTB in the right proximal ulna. A CT-guided needle biopsy revealed rapid growth and high 18F-FDG uptake, though no histological signs of malignancy were present. Given that the lung lesion was not surgically removable, Denosumab was administered. After 18 months of initial treatment with Denosumab, there were no symptoms or signs of tumor growth. While the long-term efficacy and safety of Denosumab remain to be determined, the authors posit that it may represent a viable treatment option for patients with non-resectable pulmonary GCTB.

Zhang et al. have made a notable advancement in the local drug delivery for bone tumors through the innovative use of a composite scaffold made of poly (3-hydroxybutyrate-co-3-hydroxyvalerate) (PHBV), polyethylene glycol (PEG), and melatonin. This method presents a promising avenue for enhancing bone cancer treatment by minimizing the systemic side effects of chemotherapy.

In another study by Zhang et al., nanoparticles are seen as an emerging drug delivery system that could improve the precision and efficacy of osteosarcoma treatment, reduce the side effects associated with chemotherapy, and enhance patient outcomes through targeted delivery strategies. Although the clinical application of nanoparticles in osteosarcoma treatment is still under investigation, their potential and future prospects are significant and are anticipated to play a crucial role in future clinical practice.

Zhu et al. reviewed the potential of nanomedicine to enhance immunotherapy by targeting bone marrow cells within the tumor microenvironment, highlighting the innovative thinking that is propelling the field forward. By modulating the immunosuppressive effects of bone marrow cells, these therapies aim to maximize the immune system’s potential to combat cancer cells, marking a new frontier in the treatment of bone and soft tissue tumors.

Hou et al. emphasized the use of engineered biomaterials to mitigate chemotherapy-related cardiac toxicity, showcasing innovative solutions to improve the safety and tolerability of cancer treatments. These biomaterials, by delivering chemotherapy drugs directly to the tumor site, can reduce systemic exposure and toxicity, making chemotherapy a more feasible option for patients with bone and soft tissue tumors.

Takeshima et al. stressed the critical importance of fertility preservation in young patients undergoing treatment for malignant bone and soft tissue tumors. With the increasing use of alkylating agents, which are known to cause gonadal toxicity, the preservation of sperm prior to the initiation of treatment is a crucial consideration in the comprehensive care of these patients. This aspect of care is vital as it pertains to long-term quality of life and the potential for family planning post-treatment.

Osumi et al. study on the role of proteoglycan synthesis genes in osteosarcoma stem cells offers valuable insights into cellular mechanisms that could be targeted for therapy. Understanding the pathways that govern the self-renewal and differentiation of osteosarcoma stem cells is essential for developing targeted therapies to effectively counteract tumor recurrence and metastasis. These therapies are designed to disrupt specific molecular pathways driving tumor growth, offering a more personalized approach to treatment.

Zhou et al. reviewed the role of hypoxia-inducible factor-1 alpha (HIF-1α) in osteosarcoma metastasis, providing a comprehensive summary of the molecular mechanisms involved. HIF-1α, a key regulator of cellular response to hypoxia, is increasingly recognized as a potential therapeutic target for metastatic osteosarcoma. Disrupting the complex signaling pathways regulated by HIF-1α may inhibit the onset and progression of metastasis, offering renewed hope for patients with advanced disease.

Yang et al. emphasized that the rich vascular system of bone marrow provides favorable conditions for tumor cell growth, and circulating tumor cells can form micrometastases in the early stage of disease and promote metastasis through molecular mechanisms. Symptomatic bone marrow metastasis is associated with severe myelosuppression and poor prognosis. Common symptoms include anemia, thrombocytopenia, and abnormal coagulation. The effect of myelosuppression should be considered in treatment.

In summary, this Research Topic’s articles highlight innovative research and clinical progress in bone and soft tissue tumor treatments. Advances in targeted therapies, nanotechnology for drug delivery, and immunotherapy illustrate significant progress. With a deeper understanding of tumor biology, integrating these approaches should improve patient outcomes. Personalized medicine is a continuous pursuit, and these studies are key steps towards it. We hope this Topic encourages further research and interdisciplinary collaboration to develop advanced treatments for these tumors.

